# Curious Vascular Tumor

**DOI:** 10.1155/2012/542594

**Published:** 2012-11-26

**Authors:** Faissal Jghaimi, Hasna Baallal, Anas Fakhri, Hanane Rais, Noama Karbout, Nadia Akhdari, Said Amal, Badia Belaabidia

**Affiliations:** ^1^Department of Pathology, Mohammed VI University Hospital, Marrakech, Morocco; ^2^Department of Dermatology, Mohammed VI University Hospital, Marrakech, Morocco

## Abstract

*Introduction*. Sinusoidal hemangioma is a rare variant of acquired cavernous hemangioma predominantly occurring in females. Very few case reports have been described in the literature. *Case Report*. We present a case of a 46-year-old woman who noticed a slowly growing, cutaneous nodule on the left breast. Local excision of the lesion was performed and histology allowed to find a sinusoidal hemangioma. No recurrence was noticed. *Conclusion*. The very few reports of such a lesion in the literature reflect either rarity of such lesions or unfamiliarity of this subset among the pathologists.

## 1. Introduction

Sinusoidal hemangioma (SH) was first described by Calonje and Fletcher [1] in 1991. They described this acquired benign vascular lesion as a rare subset of cavernous hemangioma. The lesions, occurring mostly in females, were solitary, deep dermal, or subcutaneous lobular vascular nodules histologically composed of dilated intercommunicating vascular channels lined by a single layer of endothelial cells with occasional pseudopapillae formation. They may have many clinical aspects, and microscopically some cases can mimic well-differentiated angiosarcoma. Although SHs are very rare, their clinicopathological characteristics are important to be recognized in order to avoid diagnostic pitfalls. We are reporting such a case occurring in a 46-year-old woman and briefly reviewing the literature 

## 2. Case Report

**Figure 1:**
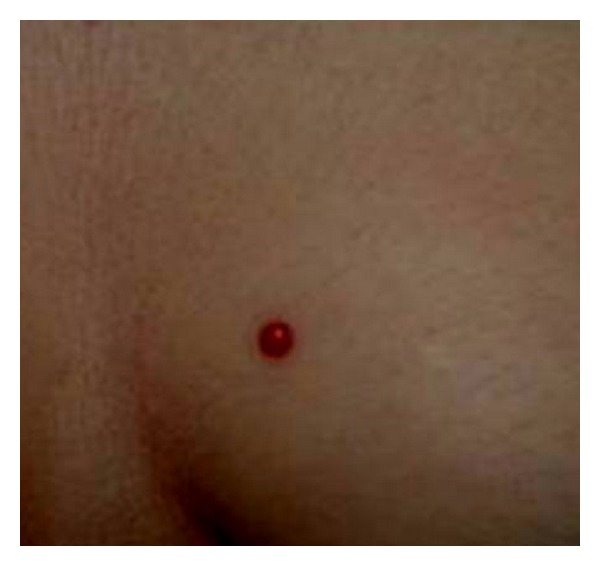
Breast cutaneous nodule with purplish discoloration of the overlying skin.

**Figure 2:**
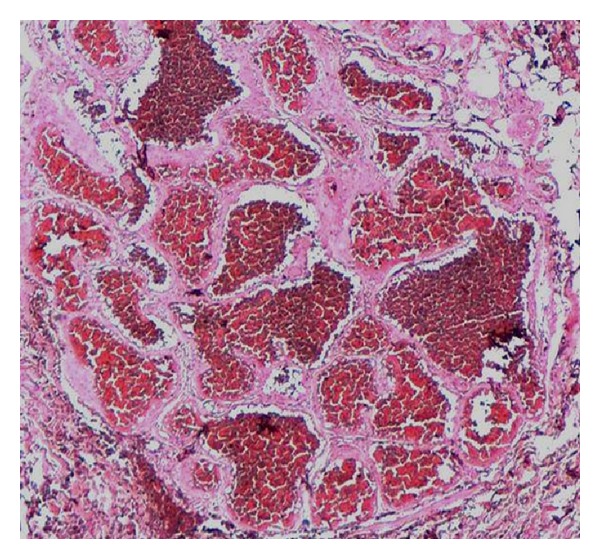
Well-circumscribed tumor located in the deep dermis and the subcutaneous tissue.

**Figure 3:**
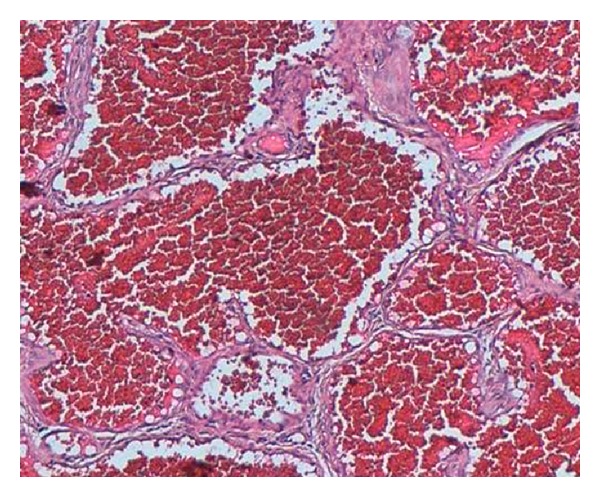
Sinusoidal patterns and “back-to-back” arrangements with pseudopapillary growth.

**Figure 4:**
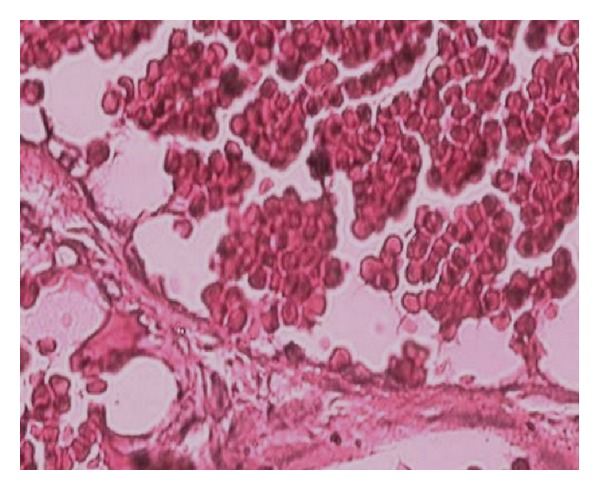
Single layer of flattened endothelial cells without nuclear atypia or mitoses.

A 46-year-old woman had a painless, slowly growing, cutaneous nodule on the left breast with a duration of 6 months. Clinical examination showed a nontender, firm, slightly raised 1 cm cutaneous nodule with purplish discoloration of the overlying skin (Figure 1). There was no other systemic disease. The patient underwent an excisional biopsy of the lesion. Gross examination showed a subcutaneous, well-demarcated, and lobulated mass measuring 1 cm. Cut surface was spongy, hemorrhagic, and firm. Microscopically, the tumor was well circumscribed, located in the deep dermis and the subcutaneous tissue (Figure 2), and was composed of multiple lobules of greatly dilated, thin walled ramifying and anastomosing vascular channels (Figure 3), demonstrating a characteristic sinusoidal appearance with area of pseudopapillary pattern (Figure 3). Closely packed intercommunicating cavernous vessels filled with blood and lined by a single layer of flattened endothelial cells were observed (Figure 4). There was no significant nuclear atypia or mitoses of the endothelial cells. Six months after excision, there was no sign of recurrence.

## 3. Discussion

In 1991, Calonje and Fletcher [1] described 12 patients with a vascular tumor that they considered to be an adult variant of cavernous hemangioma. They proposed that the entity be called “sinusoidal hemangioma.” Only few cases have been reported since Calonje and Fletcher described this entity [2–7]. It appears that SHs are uncommon. Nonetheless, the apparent rarity of reported cases might reflect the fact that, despite having somewhat atypical features, most cases are probably diagnosed as cavernous hemangiomas or simple hemangioma in the absence of better name. SHs pathogenesis is for the most part unknown, but is speculated that may involve abnormalities of the vasculogenesis and angiogenesis. Clinical features of this hemangioma comprise an acquired solitary lesion in adults, predominance on females. The most usual location of the lesions is on the upper extremities, shoulders, breast, and anterior abdominal wall. The lesions are painless and slow growing over a period ranging from 3 weeks to 20 years before they were diagnosed. SH has been estimated to be a relatively small tumor. Grossly, the tumor varied in size from 1 to 3.5 cm. However, the largest reported lesion measured 11, 5 cm in diameter [5]. They were well circumscribed, hemorrhagic, and blue red with spongy cut surface. Microscopically, the deep dermis and the subcutaneous tissue showed the presence of a lobulated mass composed of thin-walled, intercommunicating vascular channels arranged in a sinusoidal pattern, and the pseudopapillary pattern in which crosscut vessel walls are lined by flattened endothelial cells showing focal pleomorphism and hyperchromatic nuclei. The dilated vessels have an almost “back-to-back” arrangement without much intervening stroma. Thrombosis of vascular channels occurs in a proportion of cases. This can lead to intravascular papillary endothelial hyperplasia and calcification may result. Cavernous hemangioma differs principally in its tendency to appear in childhood, be of a larger size, and present on the upper body. Histologically, in contrast to sinusoidal hemangioma, a cavernous lesion has a nonlobular, poorly demarcated structure and no pseudopapillary appearance. Focal nuclear pleomorphism combined with a pseudopapillary growth pattern may lead to a misdiagnosis of well-differentiated angiosarcoma [1, 6]. These atypical features become a particular problem in evaluating a lesion from the breast, because of the well-recognized tendency mammary angiosarcoma to adopt a very bland appearance, which belies its aggressive behaviour. However, angiosarcomas of the breast are generally intraparenchymal lesions. Ki67 index can be used as a useful diagnostic to distinguish benign from malignant vascular lesions. The histopathological pattern of interconnected blood vessels with the presence of pseudopapillae has been found, although focally, in other vascular tumors, such as spindle cell hemangioma and juvenile hemangioma. 

The sinusoidal hemangioma is cured by simple local excision. Evolution of sinusoidal hemangiomas is different in different reports. Most authors, including Calonje and Fletcher [1], both for the cases investigated a benign evolution of lesions after surgical excision. Followup of the patients has revealed no tendency toward either local recurrences or metastasis [1]. In contrast, Enjolras et al. [2] four such cases shall congenital or childhood-onset and aggressive clinical course and prolonged. 

## 4. Conclusion

SH is a variant of acquired cavernous hemangioma that occurs mostly in women. Very few reports of such a lesion are described in the literature. The present case that we have reported appears to be a typical sinusoidal hemangioma clinically and histologically as described by the original authors. Local excision is the accepted therapy.
